# Enhanced AAV production via rational design of a novel pHelper vector integrated with HSV-1 helper genes

**DOI:** 10.1016/j.synbio.2025.12.018

**Published:** 2026-01-15

**Authors:** He Ren, Jianqi Nie, Zichuan Song, Wanting Mo, Yankun Yang, Zhonghu Bai

**Affiliations:** a School of Biotechnology and Key Laboratory of Industrial Biotechnology of Ministry of Education, Jiangnan University, Wuxi, 214122, China; b National Engineering Research Center of Cereal Fermentation and Food Biomanufacturing, Jiangnan University, Wuxi, 214122, China; c Key Laboratory of Carbohydrate Chemistry and Biotechnology, Ministry of Education, School of Life Sciences and Health Engineering, Jiangnan University, Wuxi, 214122, China; d Zhengzhou University of Technology, Zhengzhou, 450044, China

**Keywords:** Adeno-associated virus production, Vector design, pHelper vector, Triple-plasmid transfection, HEK293

## Abstract

Adeno-associated virus (AAV) vectors are widely used in gene therapy owing to their safety, stability, and broad tissue tropism. However, current plasmid-based AAV manufacturing platforms suffer from low yield and high manufacturing cost, limiting their scalability for clinical and commercial applications. Rational engineering of pHelper vector offers an effective strategy to enhance AAV production. In this study, we engineered a novel helper vector (UL12-ICP22-miniHelper) by integrating *UL12* and *ICP22* genes from herpes simplex virus type 1 (HSV-1) into a size-reduced pHelper backbone (mini-pHelper) with partial deletion of *E2a* and *E4* regions. In a triple-plasmid transfection system, UL12-ICP22-miniHelper increased AAV5 vector yield from 1.35 × 10^11^ to 2.85 × 10^11^ vg/mL (2.11-fold) without altering the proportion of full capsids. Enhanced productivity was also observed across multiple serotypes, with increases of 2.24-fold for AAV1, 1.54-fold for AAV2, 1.88-fold for AAV6, and 2.03-fold for AAV9, while maintaining transduction efficiency. Mechanistic analysis indicated that the improved productivity was associated with elevated viral genome replication and increased Rep/Cap protein expression. Collectively, these results demonstrate that the novel UL12-ICP22-miniHelper provides a broadly applicable and cost-effective strategy for improving AAV vector manufacturing in both clinical and industrial applications.

## Graphical abstract

**Figure:**
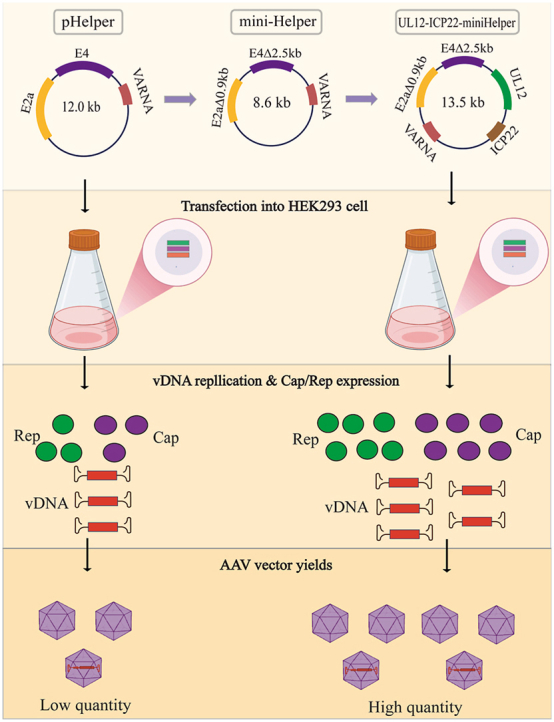

## 1 Introduction

Adeno-associated virus (AAV) vectors have emerged as a leading platform for gene therapy, owing to their capacity for long-term transgene expression [1,2], efficient transduction of both dividing and non-dividing cells, and low immunogenicity [[3], [4], [5]]. Several AAV-based therapies have received regulatory approval, including Luxturna® for retinal dystrophy, Zolgensma® for spinal muscular atrophy, and Hemgenix® for hemophilia B [[6], [7], [8], [9], [10]]. Despite these advancements, current AAV manufacturing processes remain time-consuming and costly, posing substantial barriers to clinical scalability and limiting the commercial accessibility of approved therapies [[11], [12], [13], [14]].

For research and preclinical applications, the triple‐plasmid transfection system in HEK293 cells remains the most widely used methodology for AAV production [[15], [16], [17]]. This system consists of: (1) a plasmid encoding AAV *Rep* and *Cap* genes; (2) a plasmid carrying recombinant AAV genome by inverted terminal repeats (ITRs); and (3) a helper plasmid (pHelper vector) expressing adenoviral helper genes [18]. This modular approach enables scalable AAV production while eliminating biosafety concerns associated with live adenovirus [[19], [20], [21]]. The pHelper vector expresses adenoviral genes including *E2a*, *E4orf6*, and *VA RNA*, which provide essential helper functions and enhance AAV DNA replication. However, AAV vector yields from this plasmid-based system are consistently approximately 10-fold lower than those achieved through co-infection with wild-type adenovirus [22], highlighting the need to optimize the pHelper plasmid to enhance AAV production efficiency in HEK293 cells.

In addition to adenovirus, several other viruses—including herpes simplex virus type 1 (HSV-1) [23], human papillomavirus type 16 (HPV-16) [24], and human bocavirus 1 (HBoV1) [25]—have been identified as capable of promoting AAV replication. The functional roles of helper factors derived from these viruses have been well characterized and are summarized in a previous report [26]. The primary functions of these factors are promoting AAV genome replication and supporting the expression of AAV Rep proteins [25,27]. Among them, the *NP1* and *NS2* genes from HBoV1 have been incorporated into the pHelper vector to successfully enhance AAV vector production [28]. In addition, recent studies have indicated the potential of an HSV-based platform for efficient AAV vector manufacturing, thereby identifying HSV-1 genes as potential enhancers for improving AAV production [29,30]. Significant progress has also been made in reducing the size of the commercial pHelper to improve transfection efficiency. For example, Jiten Doshi et al. and Laura van Lieshout et al. reported that a minimal adenoviral helper plasmid–generated by deleting 900 bp from the *E2a* region and removing open reading frames (ORFs) 1–4 from the *E4* gene–was capable of producing high-titer AAV in HEK293 cells [31,32]. Collectively, these findings suggest that rational engineering of the pHelper is a promising strategy to enhance AAV production efficiency.

In this study, we first systematically screened several potential helper factors from HSV-1, HBoV1, and HPV-16, and identified that the HSV-1-derived *UL5*, *UL8*, *UL12*, and *ICP22* genes significantly enhance AAV production efficiency. We then constructed a size-reduced pHelper plasmid (mini-pHelper) with deletion of 900 bp in the *E2a* intron and ORF 1–4 from the *E4* region, which is capable of maintaining AAV vector yield. Based on these findings, we engineered a novel helper plasmid (UL12-ICP22-miniHelper) by integrating *UL12* and *ICP22* into this mini-pHelper backbone. Notably, this novel UL12-ICP22-miniHelper plasmid significantly enhanced AAV vector yield in the triple-plasmid transfection system without compromising packaging efficiency or transduction potency.

## 2 Methods

### 2.1 Plasmid construction

The pAAV-RC1 (Plasmid #112862), pAAV-RC2 (Plasmid #104963), pAAV-RC5 (Plasmid #104964), pAAV-RC6 (Plasmid #110770), and pAAV-RC9 (Plasmid #112865) were obtained from Addgene. The pHelper was purchased from Takara, Japan. The pAAV-CMV-EGFP was purchased from ThermoFisher Scientific, USA. Viral helper factors derived from HSV-1, HPV-16, and HBoV1 were synthesized and cloned into the multiple cloning site (MCS) of the pcDNA3.1(+) plasmid backbone to construct helper factor–pcDNA3.1 vectors (GenScript, China). The mini-pHelper plasmid was generated by deleting a 0.9 kb intronic region from the *E2a* gene and a 2.5 kb fragment encompassing ORFs 1–4 from the *E4* region of the original pHelper, according to established methods [31]. Subsequently, selected viral helper factors were inserted into the pHelper or mini-pHelper backbone to construct novel helper plasmids.

### 2.2 AAV vector production

Suspension-adapted HEK293 cells were seeded at a density of 1.3 × 10^6^ cells/mL in 125 mL shake flasks (Jet Bio-Filtration, China) containing 30 mL of BalanCD (Irvine Scientific, USA) supplemented with 4 mM l-glutamine, prior to transfection. For the triple-plasmid transfection system, cells were transfected with 0.70 μg/mL total DNA using equimolar ratios of pHelper, pAAV-CMV-EGFP and pAAV-RC. For the four-plasmid transfection system, cells were transfected with 0.90 μg/mL total DNA using equimolar ratios of pHelper, pAAV-CMV-EGFP, pAAV-RC, and helper factor–pcDNA3.1 constructs. PEImax (Polysciences, USA) was used as transfection reagent at a DNA: PEI mass ratio of 1:1.4. The control group for four-plasmid transfection system was a pcDNA3.1(+) vector instead of the helper factor–pcDNA3.1 constructs. Cell suspensions were collected at 24 h post-transfection (hpt) for gene expression analysis, and at 72 hpt to determine vector genome (VG) titers and to characterize AAV genome encapsulation within the capsid.

### 2.3 Viral RNA extraction and analysis of gene expression

Total RNA was extracted from cell suspensions collected at 24 hpt according to the manufacturer's protocol (QIAGEN, Germany). Subsequently, 5 μL of the RNA eluent was used for cDNA synthesis with the Superscript IV First-Strand Synthesis System (Invitrogen, USA). Equal amounts of cDNA were used as templates to quantify viral factor expression levels using TaqMan Universal PCR Master Mix (ThermoFisher Scientific, USA) in a StepOnePlus Real-Time PCR System (Applied Biosystems, USA). Primer sequences are provided in Supplementary Table S1. GAPDH was detected as a housekeeping reference gene.

### 2.4 Quantification of helper plasmid, replicated viral DNA, and AAV vector by qPCR

To quantify copy numbers of helper plasmid in HEK293 cells, total cellular DNA was extracted at 24 hpt using the FastPure Cell/Tissue DNA Isolation Kit (Vazyme, China), following the manufacturer's instructions. The extracted DNA was used as the template for qPCR with primers targeting the pHelper backbone. The amount of replicated viral DNA was measured following previously established methods [33,34]. Cell pellets were collected at 72 hpt and total DNA was extracted using the FastPure Cell/Tissue DNA Isolation Mini Kit (Vazyme, China). The copies of the pAAV-GFP plasmid were quantified using primers specific to the plasmid backbone. Total GFP gene copies were measured using primers targeting the GFP coding region. The amount of replicated viral DNA was calculated by subtracting the plasmid-derived GFP copies from the total GFP gene copies. To determine VG titers, cell suspensions harvested at 72 hpt were diluted in TE buffer containing 1 % Pluronic F-68 and treated with 1 U TURBO DNase (Merck, USA) at 37 °C for 30 min to degrade unpackaged DNA, followed by heat inactivation at 75 °C for 10 min. Samples were then incubated with 1 % SDS and heated to 95 °C to fully denature the AAV capsids. VG quantification was performed by qPCR using EGFP-specific primers, as previously described. Primer sequences are provided in Supplementary Table S1.

### 2.5 Western blotting for measurement of intracellular proteins

Cell cultures were harvested and lysed using RIPA buffer (ThermoFisher Scientific, USA) according to the manufacturer's protocol. Equal amounts of total protein extracts were loaded to each lane of 10 % tris-glycine gels and subsequently transferred to PVDF membrane for detection of Rep and Cap proteins using the iBlot transfer system (Bio-Rad, UK). The blotted membranes were probed with anti-Rep antibody (1:500, Progen, Germany), anti-AAV VP1/VP2/VP3 monoclonal antibody (1:2000, Progen, Germany) and anti-β-actin antibody (1:1000, Proteintech, China). HRP-conjugated secondary antibodies (anti-mouse IgG, Cell Signaling Technology, USA) were used at a 1 in 5000 dilution. The chemiluminescent signal was detected with Clarity Western ECL reagent (Bio-Rad Laboratories, UK) using iBright CL1500 system (ThermoFisher Scientific, USA).

### 2.6 AAV transduction assay

For quantification of AAV transduction in HEK293 cells, 6-well tissue culture plates were seeded with 6 × 10^5^ cells per well and incubated for 24 h in DMEM supplemented with 10 % FBS. AAV preparations with equal titers were diluted in DPBS and added to the cells. GFP expression was assessed using either fluorescence microscopy (DP73 Microscope Digital Camera, Olympus, Japan) or flow cytometry with LSRFortessa X-20 Cell Analyzer (BD Biosciences, USA) at 72 h post-transduction.

### 2.7 Characterization of AAV genome encapsulation in capsids

Cells were harvested by centrifugation at 72 hpt and resuspended in lysis buffer consisting of 20 mM Tris (pH 8.0), 2 mM MgCl_2_, 200 mM NaCl. The suspension was subjected to three freeze-thaw cycles to release AAV vectors, and then treated with 200 U/mL Benzonase DNase (Millipore, USA) for 1 h, followed by collecting the supernatant to a new Eppendorf tube. The clarified cell lysates were loaded on an affinity chromatography (Capture Select AAVX, ThermoFisher Scientific, USA) in order to purify AAV vector, as stated previously [35]. The purified AAV vector was then buffer exchanged into10 mM bis-tris propane (BTP, pH 9.0) before injected into the column. AAV full/empty particle analysis was conducted using an anion-exchange monolith column (CIMac™ Analytical Monoliths 0.1 mL, Sartorius, Germany) coupled to Alliance HPLC system (Waters, USA). The buffer compositions were as follows: (A) 10 mM BTP, pH 9.0; (B) 10 mM BTP, 50 mM MgCl_2_, 500 mM NaCl, pH 9.0. Full and empty capsids were separated by anion-exchange chromatography according to the manufacturer's instructions. The absorbance at 280 nm was used to quantify the percentage of full capsids, calculated as

$$\%Full=\frac{Peak\;Area\;\left[Full\;Capsid\right]}{Peak\;Area\;\left[Full\;Capsid\right]+Peak\;Area\;\left[Empty\;Capsid\right]*9}\times 100$$

where Peak area [Full Capsid] and Peak area [Empty Capsid] represent peak area of full capsid and empty capsid, respectively. The number 9 represents the extinction coefficient of the empty capsid peak at 280 nm, which was determined using an AAV5 standard of known concentration.

## 3 Results

### 3.1 Evaluation of AAV vector yield improvement of viral helper factors via four-plasmid transfection

**Fig. 1:**
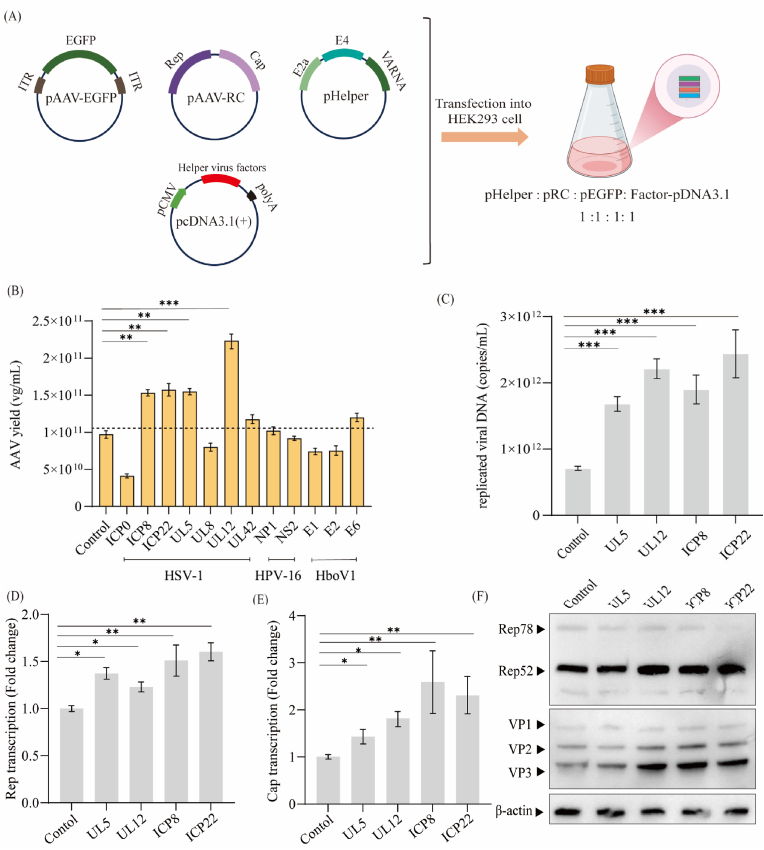
**Evaluation of viral helper factors to improve AAV yield via four-plasmid transfection.** (A) Schematic diagram of AAV vector production via four-plasmid transfection system. Equimolar ratio of pHelper, pAAV-RC, pAAV-EGFP and viral helper factor-pcDNA3.1 (pcDNA3.1 carrying helper virus factors) were co-transfected into HEK293 cells for AAV5 production. (B) AAV5 vector yield in HEK293 cells via four-plasmid transfection. VG titers in cell suspensions were measured at 72 hpt using EGFP-specific qPCR. Data were calculated from three biological replicates. Statistical was analyzed by one-way ANOVA, ∗∗*p* < 0.01, ∗∗∗*p* < 0.001. (C) Total replicated viral DNA measured at 72 hpt. (D–E) Relative transcript levels of Cap and Rep measured at 24 hpt using GAPDH as the reference gene. Transcript levels are presented as fold-change relative to the control group. Data were calculated from three biological replicates. One-way ANOVA analysis indicates a statistically significant difference between control group with various helper virus factors group (∗*p* < 0.05, ∗∗*p* < 0.01, ∗∗∗*p* < 0.001). (F) Western blot analysis of AAV Rep and Cap protein expression at 72 hpt using four-plasmid transfection. β-actin was detected as a loading control.

To preliminarily evaluate whether viral helper factors can enhance AAV production, we synthesized 12 helper candidates derived from HSV-1, HPV-16, and HBoV1, and cloned into the MCS site of the pcDNA3.1(+) vector, respectively. HEK293 cells were co-transfected with viral helper factor-pcDNA3.1, pHelper, pAAV-CMV-EGFP, and pAAV-RC5 (Fig. 1A). We first assessed the transcriptional activity of the viral factors. As shown in Fig. S1, all constructs exhibited strong transcriptional activity, except ICP0, which showed relatively weak transcription. The VG titers (Fig. 1B) indicated that four HSV-1 derived genes—*ICP8*, *ICP22*, *UL5*, and *UL12*—significantly improved AAV vector yield at harvest by 1.57- to 2.26-fold compared with the control group. In contrast, other helper factors exhibited no improvement or even a reduction in AAV vector yield. We then analyzed the levels of replicated viral DNA and expression of Rep and Cap proteins in groups that exhibited increased AAV vector yields (*ICP8*, *ICP22*, *UL5*, *and UL12*). Replicated viral DNA levels at 72 hpt were elevated 2.37- to 3.43-fold relative to control group (Fig. 1C), consistent with previous reports that these viral factors enhance AAV genome replication [23]. Transcriptional analysis by quantitative reverse transcription PCR (RT-qPCR) revealed varying degrees of upregulation in Rep and Cap transcripts at 24 hpt (Fig. 1D and E). Western blot analysis further confirmed the increase in Rep and Cap protein expression (Fig. 1F). Three of these viral factors, excluding *UL5*, led to an obviously increase in Rep52 and Cap protein level. These results suggest that *ICP8*, *ICP22*, *UL5*, and *UL12* enhance AAV production in the four-plasmid transfection system. To simplify the AAV production process, it is necessary to integrate these effective factors into the pHelper.

### 3.2 Integration of viral helper factors into pHelper for AAV production

**Fig. 2:**
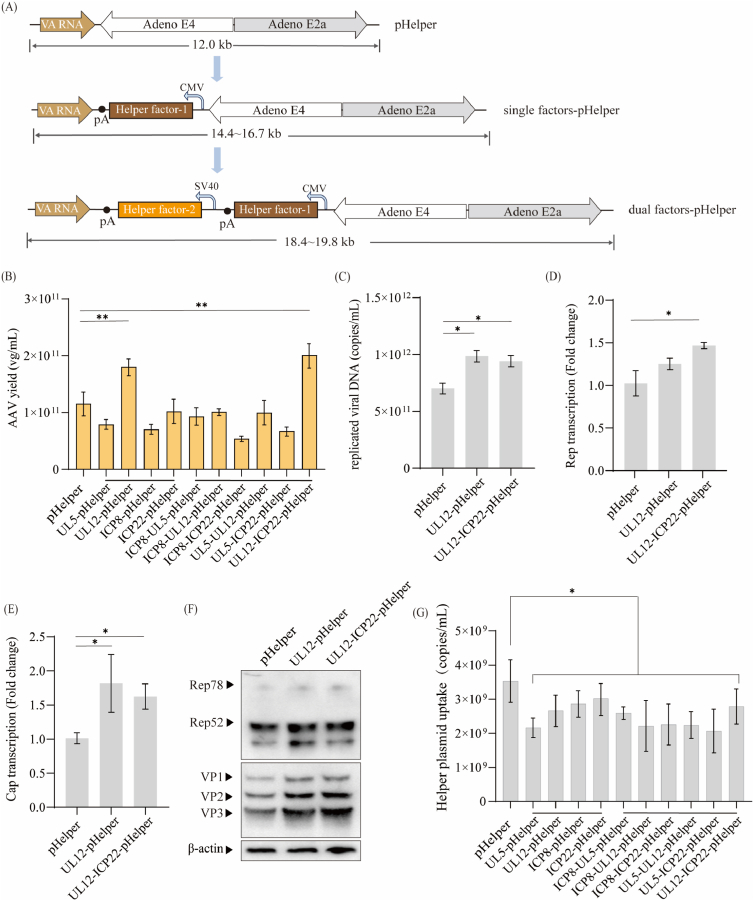
**Integration of viral helper factors into pHelper for AAV production.** (A) Schematic diagram of the engineered pHelper constructs, in which single or dual helper virus genes (UL5, UL12, ICP8, and ICP22) were inserted between the *VA RNA* and *E4* regions. (B) AAV5 vector yield in HEK293 cells co-transfected with pAAV-EGFP, pAAV-RC5, and pHelper or constructed helper plasmid. VG titers in cell suspensions were measured at 72 hpt using EGFP-specific qPCR. Data were calculated from three biological replicates. Statistical was analyzed by one-way ANOVA, ∗∗*p* < 0.01. (C) Total replicated viral DNA measured at 72 hpt. and relative transcript levels of Cap (D–E) Relative transcript levels of Cap and Rep measured at 24 hpt using GAPDH as the reference gene. Relative transcript is presented as fold-change to pHelper group. Data were calculated from three biological replicates. One-way ANOVA analysis indicates a statistically significant difference between control group with various helper virus factors group (∗*p* < 0.05). (F) Western blot analysis of AAV Rep and Cap protein expression at 72 hpt co-transfected with pAAV-EGFP, pAAV-RC5, and pHelper, UL12-pHelper or UL12-ICP22-Helper. β-actin was detected as a loading control. (G) Assessment of helper plasmid uptake per mL co-transfected with pAAV-EGFP, pAAV-RC5, and pHelper or constructed helper plasmid. Statistical significance was calculated by one-way ANOVA with Tukey's multiple comparisons, ∗*p* < 0.05.

To evaluate whether incorporation of these four helper factors into the pHelper could enhance AAV production in the conventional triple-plasmid system, we constructed 10 modified helper plasmids by inserting one or two of the selected viral genes between the *VA RNA* and *E4* regions (Fig. 2A). Each constructed helper plasmid was co-transfected with pAAV-CMV-EGFP and pAAV-RC5 into HEK293 cells at equimolar DNA concentrations. Among these modified helper plasmids, only UL12-pHelper and UL12-ICP22-pHelper increased AAV vector yield by 1.60-fold and 1.72-fold, respectively, compared to the control (Fig. 2B). In addition, these two constructs also exhibited elevated replicated viral DNA (1.40- and 1.34-fold, respectively; Fig. 2C) and enhanced Rep and Cap expression (RT-PCR and Western blot analyses, Fig. 2D–F). However, the improvements in replicated viral DNA and Rep and Cap expression were smaller than those obtained in the four-plasmid system. We speculated that this discrepancy might be attributed to the increased size of the modified helper plasmids, which expanded from 12.0 kb to 19.8 kb. To test this hypothesis, we quantified cellular uptake of helper plasmid after transfection and the details about plasmid size, plasmid uptake, and AAV yield are shown in Table S2. These results revealed that the plasmid uptake decreased when modified helper plasmids with larger size were used (Fig. 2G and Table S2). These findings suggest that reducing plasmid size may improve transfection efficiency and thereby enhance AAV production.

### 3.3 A mini-pHelper containing deletions within the *E4* and *E2a* genes maintained AAV productivity

**Fig. 3:**
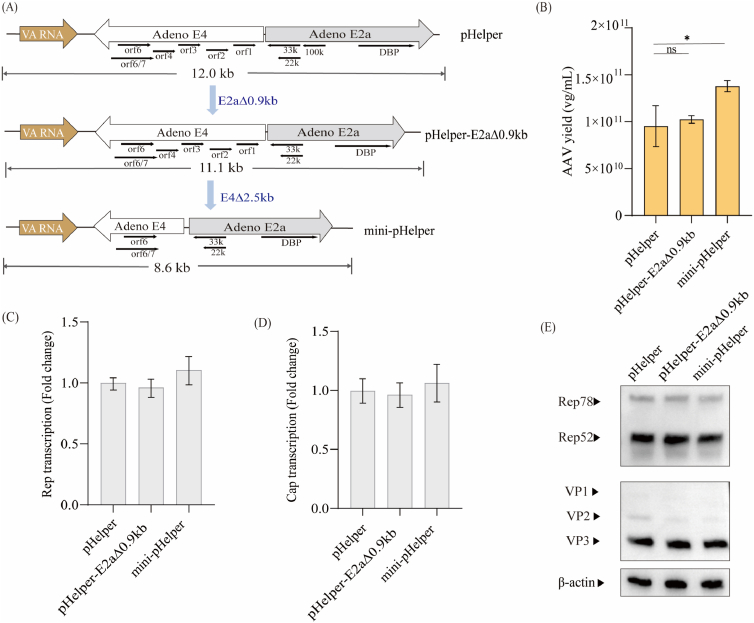
**A mini-pHelper containing deletions within the*****E4*****and*****E2a*****genes maintained AAV productivity.** (A) Schematic diagram of the mini-pHelper plasmid construction by deleting 0.9 kb of the *E2a* gene and 2.5 kb of the *E4* gene. Black arrows indicate the direction and length of protein coding sequence in the plasmid. (B) AAV5 vector yield in HEK293 cells co-transfected with pAAV-EGFP, pAAV-RC5, and pHelper, pHelper-E2aΔ0.9 kb or mini-pHelper. VG titers in cell suspensions were measured at 72 hpt using EGFP-specific qPCR. Data were calculated from three biological replicates. Statistical was analyzed by one-way ANOVA, ∗*p* < 0.05. Relative transcript levels of Rep (C) and Cap (D) using GAPDH as the reference transcript. Relative transcript is presented as fold-change to pHelper group. Data were calculated from three biological replicates. Statistical was analyzed by one-way ANOVA, ∗*p* < 0.05. (E) Western blot analysis of AAV Rep and Cap in HEK293 cells co-transfected with pAAV-EGFP, pAAV-RC5, and pHelper, pHelper-E2aΔ0.9 kb or mini-pHelper. β-actin was detected as a loading control.

Several commercially available pHelper vectors, although differing in size and specific sequence composition, consistently contain the *VA RNA*, *E4*, and *E2a* genes. However, prior studies have shown that not all of these genetic elements are essential for AAV production, as some non-essential regions can be deleted without affecting AAV production [[36], [37], [38]]. We hypothesized that integrating viral helper factors into a size-reduced pHelper could enhance the transfection efficiency. Following the reported strategy [31], we first deleted a 0.9 kb intronic sequence within the *E2a* gene while retaining the key functional elements of *DBP* and *L4-22K/33K*, thereby generating pHelper-E2aΔ0.9 kb. Then, we subsequently removed the ORFs 1–4 region (2.5 kb) of the *E4* gene, yielding the final mini-pHelper construct with a total size of 8.6 kb, which is 3.4 kb shorter than the original pHelper (Fig. 3A). Deletion of the E2a intron led to a modest reduction in DBP transcript levels, but this had no impact on AAV vector yield (Fig. 3B). In contrast, the additional deletion of *E4* ORFs 1–4 resulted in decreased E4orf6 transcript levels while increasing AAV production by 1.32-fold (Fig. 3B and Fig. S2), suggesting that removal of *E4* ORFs 1–4 may have a beneficial effect on vector production. Western blot and RT-qPCR analyses of Rep and Cap expression showed no significant differences between the pHelper and mini-pHelper (Fig. 3C–E). Interestingly, this finding contrasts with the previous report, which observed increased Cap expression using a size-reduced pHelper design [31]. We speculate that this discrepancy may be attributed to serotype-specific differences, as their study focused on AAV9, whereas our experiments primarily utilized AAV5. Nevertheless, our results demonstrate that the mini-pHelper construct effectively maintains AAV productivity while offering a reduced size that may improve transfection efficiency, particularly when incorporating the helper factors *UL12* and *ICP22*.

### 3.4 Integration of *UL12* and *ICP22* genes into the mini-pHelper further enhanced AAV vector production

**Fig. 4:**
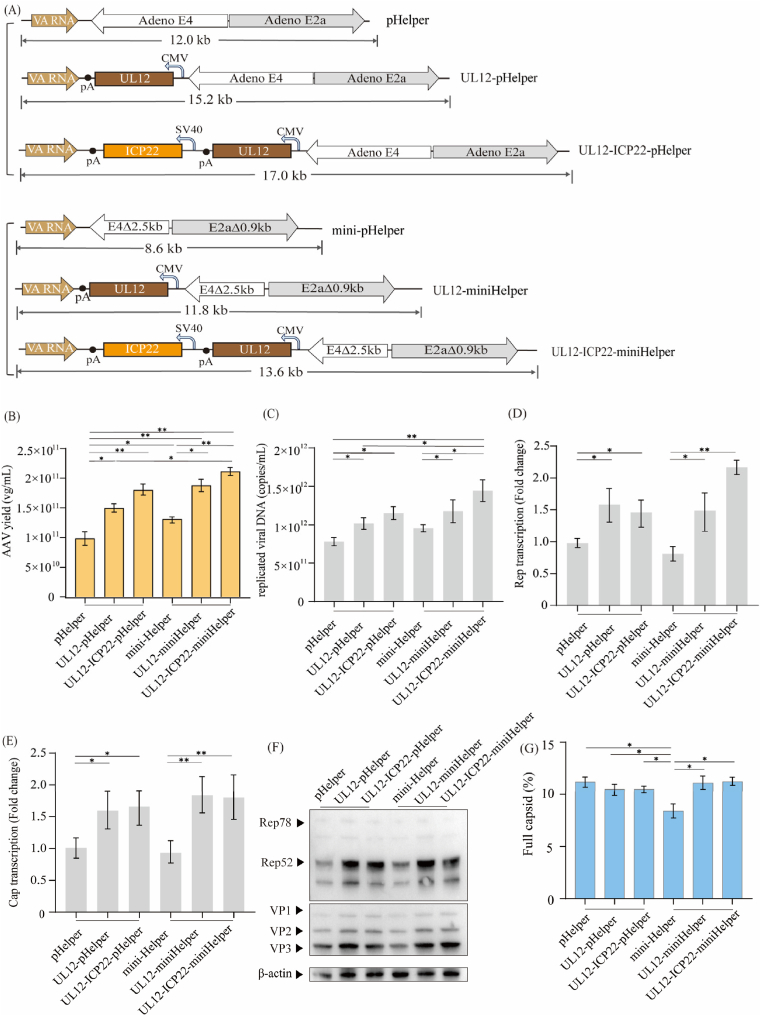
**Integration of UL12 and ICP22 genes into the mini-pHelper further enhanced AAV vector production.** (A) Schematic diagram of the engineered various constructed helper plasmid. (B) AAV5 vector yield in HEK293 cells co-transfected with pAAV-EGFP, pAAV-RC5, and constructed helper plasmid. VG titers in cell suspensions were measured at 72 hpt using EGFP-specific qPCR. Data were calculated from three biological replicates. Statistical was analyzed by one-way ANOVA, ∗*p* < 0.05. ∗∗*p* < 0.01. (C) Total replicated viral DNA measured at 72 hpt. (D–E) Relative transcript levels of Cap and Rep measured at 24 hpt using GAPDH as the reference gene. Relative transcript is presented as fold-change to pHelper group. Data were calculated from three biological replicates. Statistical was analyzed by one-way ANOVA, ∗*p* < 0.05, ∗∗*p* < 0.01. (F) Western blot analysis of AAV Rep and Cap protein expression co-transfected with pAAV-EGFP, pAAV-RC5, and constructed helper plasmid. β-actin was detected as a loading control. (G) Proportion of encapsulated genomes for AAV5 in HEK293 cells co-transfected with pAAV-EGFP, pAAV-RC5, and constructed helper plasmid. Analysis of empty to full AAV particles using anion-exchange chromatography methods on a 0.1 mL CIMmultus monolith and expressed as a fold-change compared to the pAAV-RC. Statistical was analyzed by one-way ANOVA, ∗*p* < 0.05.

After confirming that the mini-pHelper can support efficient AAV production, we next inserted the *UL12* gene or combination of *UL12* and *ICP22* genes into the mini-pHelper backbone to generate UL12-miniHelper (11.8 kb) and UL12-ICP22-miniHelper (13.6 kb), which are comparable in size to the original pHelper (12.0 kb). These modified helper plasmids, along with the original pHelper and previously constructed UL12-pHelper and UL12-ICP22-pHelper plasmids, were used in triple-plasmid transfection to produce AAV vectors, respectively. Remarkably, UL12-miniHelper and UL12-ICP22-miniHelper maintained plasmid uptake comparable to pHelper (Fig. S3) while increasing AAV vector yield by 1.82-fold and 2.11-fold, respectively (Fig. 4B). Furthermore, these mini-helper constructs achieved higher AAV yields than constructs based on the original pHelper backbone (UL12-pHelper and UL12-ICP22-pHelper; Fig. 4B), indicating that a helper plasmid with size reduced can further enhance AAV production. The mini-pHelper exhibited a slight but not statistically significant increase in replicated viral DNA, compared to the pHelper (Fig. 4C). In contrast, UL12-miniHelper and UL12-ICP22-miniHelper resulted in significantly elevated levels of replicated viral DNA, consistent with the enhancements of the UL12-pHelper and UL12-ICP22-pHelper (Fig. 4C). Furthermore, integration of UL12 and ICP22 into the mini-pHelper significantly enhanced Rep and Cap expression at both the transcriptional and protein levels (Fig. 4D–F). As shown in Fig. 4G and Fig. S4, full capsids percentage was relatively similar across groups (∼11 %), except for the mini-pHelper group, which showed a significant decrease (∼8 %). We speculate that the reduced full-to-empty ratio of mini-pHelper may be attributed to the deletion of certain sequences from the original pHelper, which compromised capsid genome packaging [39]. However, the subsequent integration of *UL12* and *ICP22* likely compensated for the functions of these deleted sequences.

### 3.5 UL12-ICP22-miniHelper improves productivity for various capsid serotypes

**Fig. 5:**
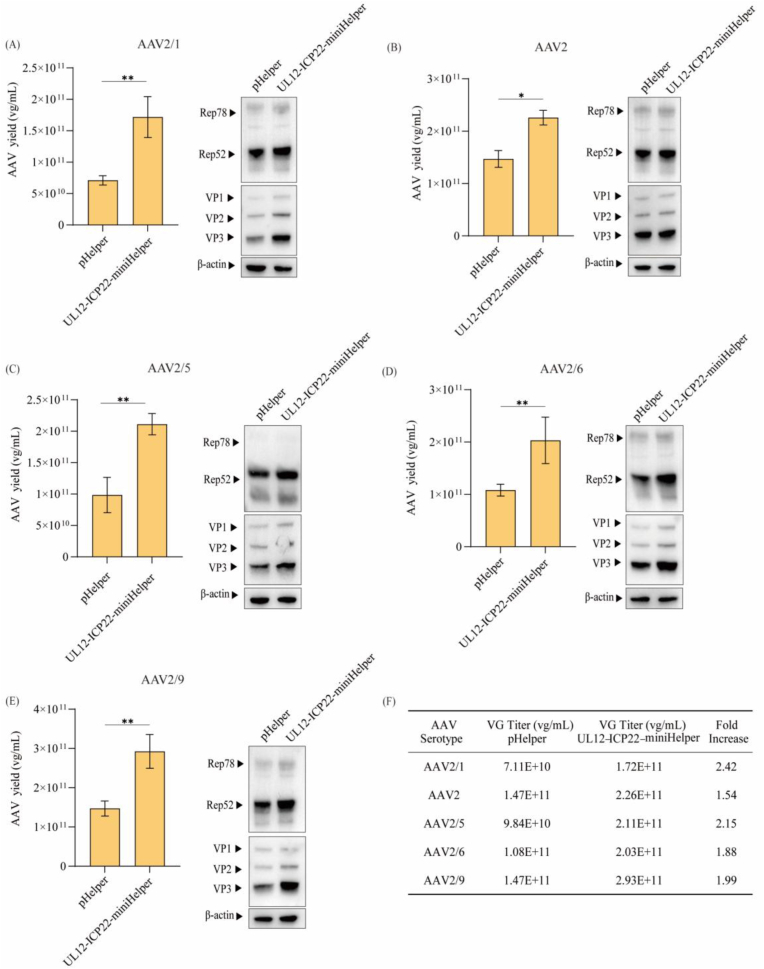
**UL12-ICP22-miniHelper improves productivity for various capsid serotypes.** Vector yields and Western blot analysis of AAV Rep and Cap expression for AAV2/1 (A), AAV2 (B), AAV2/5 (C), AAV2/6 (D), AAV2/9 (E). HEK293 cells were co-transfected with pAAV-EGFP, pRC, and pHelper or UL12-ICP22-miniHelper. VG titers in cell suspensions were measured at 72 hpt using EGFP-specific qPCR. Data were calculated from three biological replicates. Statistical was analyzed by one-way ANOVA, ∗*p* < 0.05. ∗∗*p* < 0.01. β-actin was detected as a loading control. (F) Summary of AAV vector titers across multiple serotypes using either the pHelper or the UL12-ICP22-miniHelper plasmid. Fold increases represent the ratio of VG titers obtained with UL12-ICP22-miniHelper to those obtained with pHelper.

To investigate whether the UL12-ICP22-miniHelper could enhance the production of various AAV capsid serotypes, we evaluated four additional serotypes: AAV1, AAV2, AAV6, and AAV9. VG titers elevated 1.54–2.42-fold across all serotypes compared to the original pHelper group (Fig. 5), consistent with the enhancement previously observed in AAV5. Western blot analysis further revealed that the UL12-ICP22-miniHelper enhanced Rep and Cap expression to varying degrees across multiple AAV serotypes, with the exception of AAV2 (Fig. 5B). Notably, we observed a positive correlation between the increase in VG titers and the elevated Rep and Cap expression levels. For instance, AAV1 and AAV9 exhibited the highest increases in VG titers (2.24- and 2.03-fold, respectively), accompanied by the most significant upregulation of Rep and Cap expression (Fig. 5A and E). Similarly, AAV6 production showed a 1.88-fold increase, along with a moderate elevation in Rep and Cap levels (Fig. 5D). In contrast, although AAV2 exhibited a 1.54-fold increase in VG titer, no significant change in Rep or Cap expression was detected (Fig. 5B). Collectively, these findings confirm that the UL12-ICP22-miniHelper broadly enhances AAV vector production across multiple capsid serotypes.

### 3.6 UL12-ICP22-miniHelper maintains comparable AAV transduction efficiency in vitro

**Fig. 6:**
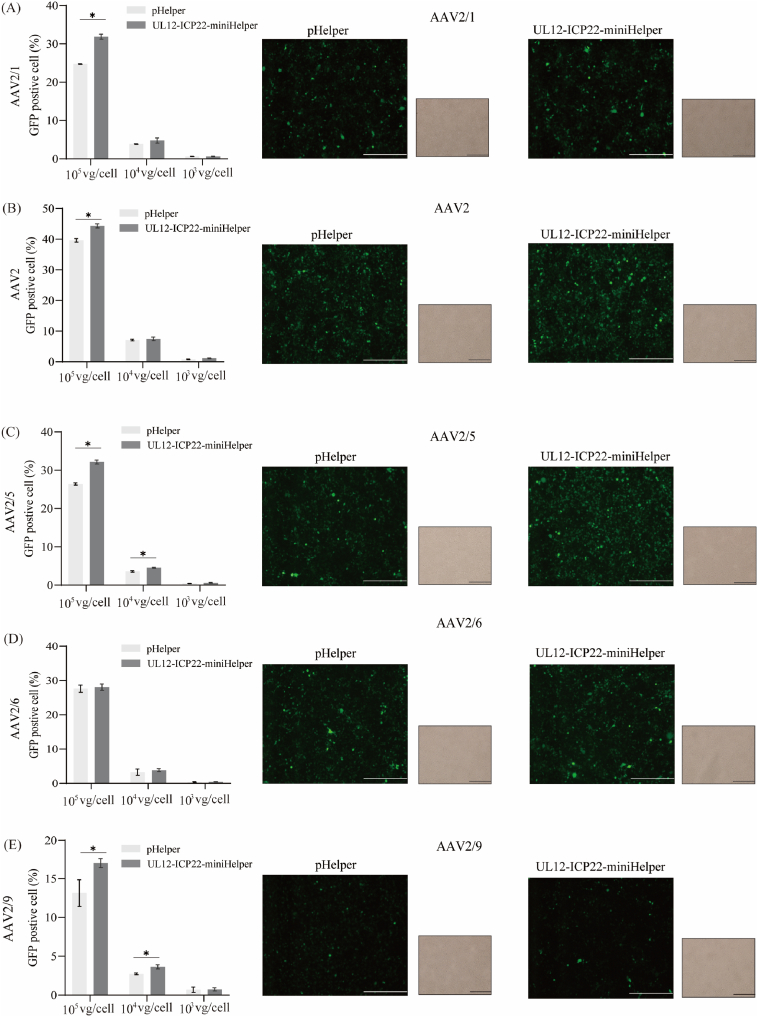
**UL12-ICP22-miniHelper maintains comparable AAV transduction efficiency in vitro.** In vitro transduction efficiency analysis of AAV vectors from various serotypes. Fluorescence microscopy images and flow cytometry of HEK293 cells transduced with AAV1-EGFP (A), AAV2-EGFP (B), AAV5-EGFP (C), AAV6-EGFP (D), and AAV9-EGFP (E). Vectors were produced via triple-plasmid transfection using original pHelper or UL12-ICP22-miniHelper. For flow cytometry, cells were transduced at 10^3^, 10^4^, or 10^5^ vg/cell. Data represent N = 3 biological replicates (mean ± SD). Statistical significance was determined by two-tailed unpaired *t*-test (∗*p* < 0.05). For fluorescence microscopy, cells were transduced at a dose of 10^3^ vg/cell and imaged 72 hpt (scale bar: 50 μm).

Given that the UL12-ICP22-miniHelper differs from the conventional pHelper by removing specific adenoviral sequences and incorporating helper factors from heterologous viruses, it is plausible that these modifications could affect the transduction efficiency of the resulting AAV vectors. To evaluate this possibility, we transduced HEK293 cells with five AAV-GFP serotypes (AAV1, AAV2, AAV5, AAV6, and AAV9) produced using either the UL12-ICP22-miniHelper or the original pHelper, and assessed transduction efficiency by quantifying the percentage of GFP-positive cells 72 h post-transduction. Overall, no significant differences in transduction efficiency were observed between the two helper vectors (Fig. 6). Although the UL12-ICP22-miniHelper group exhibited statistically significant improvements in transduction efficiency at high dose (10^5^ VG/cell, p < 0.05) for all serotypes except AAV6, no significant differences were observed at middle and low doses (10^3^ and 10^4^ VG/cell). Collectively, these results indicate that the UL12-ICP22-miniHelper supports robust AAV production without compromising transduction efficiency.

## 4 Discussion

As a member of the *Dependoparvovirus* genus, AAV requires the assistance of helper viruses or plasmids to achieve productive replication [40,41]. The commercially available pHelper expressing the adenoviral genes *E4*, *E2a*, and *VA RNA*, has been shown to support AAV production. However, several other viruses have also been identified as capable of complementing AAV replication, and the functions of their helper factors have been extensively characterized [26]. The aim of this study was to investigate whether incorporating helper factors from other viruses into the pHelper backbone could enhance AAV vector production and reduce manufacturing costs.

Using a four-plasmid transfection system (pAAV-RC, pAAV-EGFP, pHelper, and helper factor–pcDNA3.1), we identified four HSV-1-derived factors—*UL5*, *UL12*, *ICP8*, and *ICP22*—that significantly enhanced AAV vector yield (Fig. 1B). Surprisingly, helper factors from HPV-16 and HBoV1 did not improve production and even reduced yield. We hypothesize that this phenomenon may be due to functional redundancy or negative interactions between these helper factors and adenoviral components encoded by the pHelper. Further analysis revealed that several of these effective helper factors promoted viral DNA replication, consistent with their previously reported functions [23]. Additionally, the helper factors that increased VG titers also increased expression of Rep and Cap proteins (Fig. 1F), which has not been reported previously. Notably, among the four helper factors tested, UL12 produced the highest AAV vector yield, whereas its replicated genome copy number and Cap/Rep expression levels were not (Fig. 1C–F). Furthermore, a similar trend was observed for the UL12-ICP22-miniHelper (Fig. 3). Thus, AAV production does not strictly correlate with replicated genome copy number or Cap expression. As biosynthesis of AAV is a complex process, we speculate that additional steps such as genome processing, genome encapsidation, and virion release may affect AAV production as well [16,42,43].

Although co-transfection with an additional plasmid to overexpress helper factors is a simple approach, it requires extra plasmid preparation and optimization of transfection protocols. To further simplify production, we integrated helper genes into the 3′ UTR region downstream of the *VA RNA* sequence within the pHelper construct. Among the constructs tested, only UL12-pHelper and UL12-ICP22-Helper showed increases in AAV vector yield. According to Fig. 2G and Table S2, incorporation of two HSV genes into pHelper increases the plasmid size to 16.38 kb or larger. Among these constructs, UL12-ICP22-pHelper (16.38 kb) exhibited a plasmid uptake of 2.78 × 10^9^ copies/mL and an increased AAV yield of 2.04 × 10^11^ vg/mL. However, other helper plasmids with size larger than 17 kb showed marked decreased in plasmid uptake and AAV yield. These findings suggest that incorporation of multiple HSV genes into the pHelper may be counterproductive if the overall plasmid size exceeds a certain threshold (approximately 17 kb under our conditions).

To address this issue, we constructed a mini-pHelper and subsequently generated the UL12-ICP22-miniHelper construct (13.6 kb) by inserting the *UL12* and *ICP22* genes into the mini-pHelper backbone, thereby maintaining size comparable to the original pHelper (12.0 kb). Although the mini-pHelper exhibited reduced expression of E4orf6, it did not negatively affect AAV production (Fig. 3B and Fig. S2). This may be due to the fact that E4orf6 is redundant—or even detrimental for AAV manufacturing, as suggested by previous studies [32], implying that further deletion of E4orf6 could be a feasible strategy to reduce plasmid size.

The UL12-ICP22-miniHelper enhanced AAV production across multiple serotypes, with yields increased by 1.54- to 2.42-fold compared to the original pHelper (Fig. 5). This improvement is likely attributable to the functions of UL12 and ICP22. Specifically, UL12 is known to promote AAV genome replication through its nuclease activity, which may facilitate viral DNA processing and synthesis [44,45]. In addition, ICP22 has been reported to modulate host RNA polymerase activity, thereby enhancing the transcription and expression of AAV Rep and Cap proteins [46]. The E4 region has been reported to influence AAV vector preparations and to promote the release of viral particles from cells into the supernatant [47]. In particular, E4orf6 has been shown to enhance AAV2 replication by promoting second-strand synthesis [26]. As recent studies reporting that elimination of E4 can increase AAV production [31,32], we tried to delete E4 ORFs 1–4 and then constructed the UL12-ICP22-miniHelper to further enhance AAV production. As shown in Fig. 5B, UL12-ICP22-miniHelper resulted in varying degrees of enhancement in AAV production across five AAV serotypes. The enhancement observed for AAV2 was weaker than the others. This may be attributable to serotype-dependent effects of E4 genes on total AAV production and packaging efficiency, as previously reported by Fernandes et al. [48].

Analysis of purified vector preparations revealed that the mini-pHelper slightly reduced the proportion of full capsids from approximately 11 %–8 % (Fig. 4G). This suggests that although the deleted elements do not impair Rep/Cap expression or genome replication, they do affect genome packaging functions. This may be due to the E4orf6 implicated in promoting genome stability and nuclear export [39]. Notably, the introduction of the UL12 and ICP22 genes restored the proportion of full particles. This effect may be attributed to the function of UL12, which promotes the formation of AAV genome that are suitable for packaging thus increased the number of AAV particles [23].

Rational modification of the pHelper has emerged as a promising strategy for process optimization, especially as AAV manufacturing technologies continue to evolve [[49], [50], [51]]. Our findings indicate that the rational incorporation of non-adenoviral helper factors is an effective strategy for improving AAV production. A recent report concluded that rational addition of HBoV1-derived *NP1* and *NS2* genes into the pHelper resulted in approximately a 2-fold increase in AAV2 yield compared to the traditional adenovirus-based helper system [28]. However, co-transfection of NP1 or NS2 individually with pHelper in our study failed to enhance vector yield. A plausible explanation is that the functions of these two elements are specific and only take effect when they are integrated together [52,53]. This finding also suggests that rational synergistic integration of two or more elements provides a more effective strategy for optimizing AAV production.

In summary, we have constructed a novel helper plasmid, UL12-ICP22-miniHelper, which improves the production yields of various AAV serotypes without compromising packaging efficiency or transduction potency. This improvement is serotype-dependent, and further investigation is needed to explore its application in AAV production for different serotypes.

## CRediT authorship contribution statement

**He Ren:** Writing – original draft, Investigation. **Jianqi Nie:** Writing – original draft, Investigation, Data curation. **Zichuan Song:** Validation, Data curation. **Wanting Mo:** Validation, Investigation. **Yankun Yang:** Supervision, Data curation. **Zhonghu Bai:** Supervision, Resources, Funding acquisition.

## Declaration of competing interests

The authors declare that they have no known competing financial interests or personal relationships that could have appeared to influence the work reported in this paper.

## Data Availability

The data used to support the findings of this study are available from the corresponding author upon request.
