# Supplementary material for: Enhanced AAV production via rational design of a novel pHelper vector integrated with HSV-1 helper genes
Source: Synth Syst Biotechnol. 2026 Jan 15;12:352–63. doi: 10.1016/j.synbio.2025.12.018 (PMC12830312; doi:10.1016/j.synbio.2025.12.018)
Supplement: Multimedia component 1 [file mmc1.docx]

**Supplementary Information**

**Enhanced AAV Production via Rational Design of a Novel pHelper Vector Integrated with HSV-1 Helper Genes**

**He** **Ren^1,2^, Jianqi Nie^3^*,** **Zichuan Song^1,2^, Wanting Mo^1,2^,** **Yankun Yang^1,2^, Zhonghu Bai^1,2,4^***

1. School of Biotechnology and Key Laboratory of Industrial Biotechnology of Ministry of Education, Jiangnan University, Wuxi 214122, China

2. National Engineering Research Center of Cereal Fermentation and Food Biomanufacturing, Jiangnan University, Wuxi 214122, China

3. Key Laboratory of Carbohydrate Chemistry and Biotechnology, Ministry of Education, School of Life Sciences and Health Engineering, Jiangnan University, Wuxi 214122, China

4. Zhengzhou University of Technology, Zhengzhou 450044, China

* Corresponding authors

Prof. Zhonghu Bai

Email baizhonghu@jiangnan.edu.cn; Tel: +86-510-85329306; Fax: +86-510-85329306

Dr. Jianqi Nie

Email: niejianqi@jiangnan.edu.cn; Tel: +86-510-85329306; Fax: +86-510-85329306

**Table S1. qPCR primersused for detecting viral factors (5’→3’)**

| Target | | Primer/ Probe | Sequence |
| --- | --- | --- | --- |
| rAAV | EGFP | Fwd | GAACCGCATCGAGCTGAA |
|  |  | Rev | ATCGACTTCAAGGAGGACGGCAAC |
|  |  | Probe | TGCTTGTCGGCCATGATATAG |
|  | Rep | Fwd | GGCCTCATACATCTCCTTCAAT |
|  |  | Rev | AGTCAGGCTCATAATCTTTCCC |
|  | Cap | Fwd | TCGCCACGACCAATAGGATG |
|  |  | Rev | GGTTGTTGGTCATGCCGTTC |
| Plasmid | pHelper | Fwd | CACGCCCACGAGATTAGGTT |
|  |  | Rev | GAAACTCTTGGCGGGCTTTG |
|  | pAAV-GFP | Fwd | AATAAACCAGCCAGCCGGAA |
|  |  | Rev | AGACTGGATGGAGGCGGATA |
| Ad5 | E4orf6 | Fwd | GGTCTCCCCTGCAGTG |
|  |  | Rev | AGAACCATATCCCAGGGA |
|  | E2a(DBP) | Fwd | CAAGTGGAAGCTCTGG |
|  |  | Rev | GCGGTGCTCCTCGTT |
| HSV-1 | UL5 | Fwd | GCACGAGTTCGGTAACCTCA |
|  |  | Rev | TGTAGTTTTCCGGGACGACG |
|  | UL8 | Fwd | CCGTTAACATCACCACCGGA |
|  |  | Rev | CTCCTTCTTGATCGGCGGAA |
|  | UL12 | Fwd | ATGTACAGTCTTCGGCGGTG |
|  |  | Rev | AGTAGCGAAACAGCAGGCTT |
|  | UL42 | Fwd | CATCGCACCTTCTCTGTGGT |
|  |  | Rev | AGGAAGAACTTGAGGGTGCC |
|  | ICP0 | Fwd | CGTGTGCACGGATGAGATCG |
|  |  | Rev | GGTTTTCATGCACGGGATGC |
|  | ICP8 | Fwd | GTCGTTACCGAGGGCTTCAA |
|  |  | Rev | GTTACCTTGTCCGAGCCTCC |
|  | ICP22 | Fwd | GGCCCGGAGTGTGATCTTAG |
|  |  | Rev | GATCGGTGGCATCGGAGATT |
| HPV-16 | E1 | Fwd | AAAGCGAAGACAGCGGGTAT |
|  |  | Rev | AGTCTCATGGCGCCCTTCTA |
|  | E2 | Fwd | GACCCATACCAAAGCCGTCG |
|  |  | Rev | GCTCTGATCTTGGTCGCTG |
|  | E6 | Fwd | GTGTACTGCAAGCAACAGTT |
|  |  | Rev | GGATTCCCATCTCTATATAC |
| HBoV1 | NS2 | Fwd | AATGACTGCAGACAACGCCT |
|  |  | Rev | ACTTTGCAGGTTCCACCCAA |
|  | NP1 | Fwd | CTTGCACTGCTTCGAAGAC |
|  |  | Rev | GTTGACTGAATACAGTGTAT |
| / | GAPDH | Fwd | TCGACAGTCAGCCGCATCT |
|  |  | Rev | ACTCCGACCTTCACCTTCCC |

**Table S2. Summary of plasmid size, plasmid uptake, and AAV yield for constructed helper plasmids**

| Plasmid name | Plasmid size | Plasmid uptake  (copies/mL) | AAV yield (vg/mL) |
| --- | --- | --- | --- |
| pHelper | 11.95 kb | 3.53E+09 | 1.19E+11 |
| UL5-pHelper | 15.20 kb | 2.16E+09 | 7.88E+10 |
| UL12-pHelper | 14.66 kb | 2.66E+09 | 1.88E+11 |
| ICP8-pHelper | 16.15 kb | 2.86E+09 | 7.00E+10 |
| ICP22-pHelper | 13.82 kb | 3.02E+09 | 1.04E+11 |
| ICP8-UL5-pHelper | 19.33 kb | 2.59E+09 | 9.15E+10 |
| ICP8-UL12-pHelper | 18.86 kb | 2.22E+09 | 1.01E+11 |
| ICP8-ICP22-pHelper | 17.90 kb | 2.26E+09 | 5.40E+10 |
| UL5-UL12-pHelper | 17.94 kb | 2.24E+09 | 9.93E+10 |
| UL5-ICP22-pHelper | 17.01 kb | 2.07E+09 | 6.78E+10 |
| UL12-ICP22-pHelper | 16.38 kb | 2.78E+09 | 2.04E+11 |


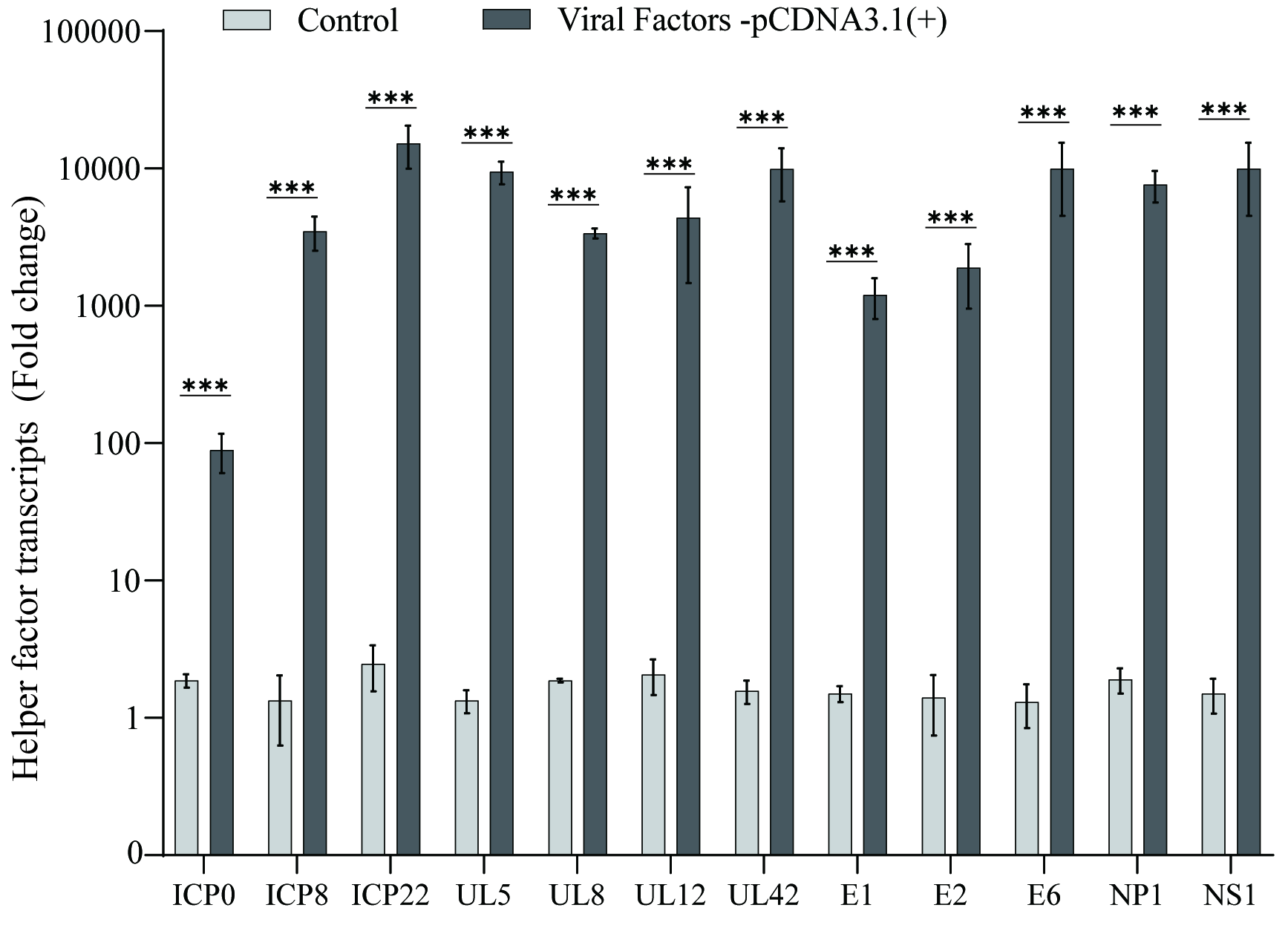


**Fig S1. Relative transcriptional activity of viral helper factors in a four-plasmid transfection system.**

HEK293 cells were transfected using a four-plasmid system, and total RNA was extracted 24 hpt for cDNA synthesis. The relative mRNA expression levels of viral helper factors were quantified by qPCR. Data were calculated from three biological replicates. Statistical was analyzed by two-tailed unpaired t-test, *** *p*< 0.001.


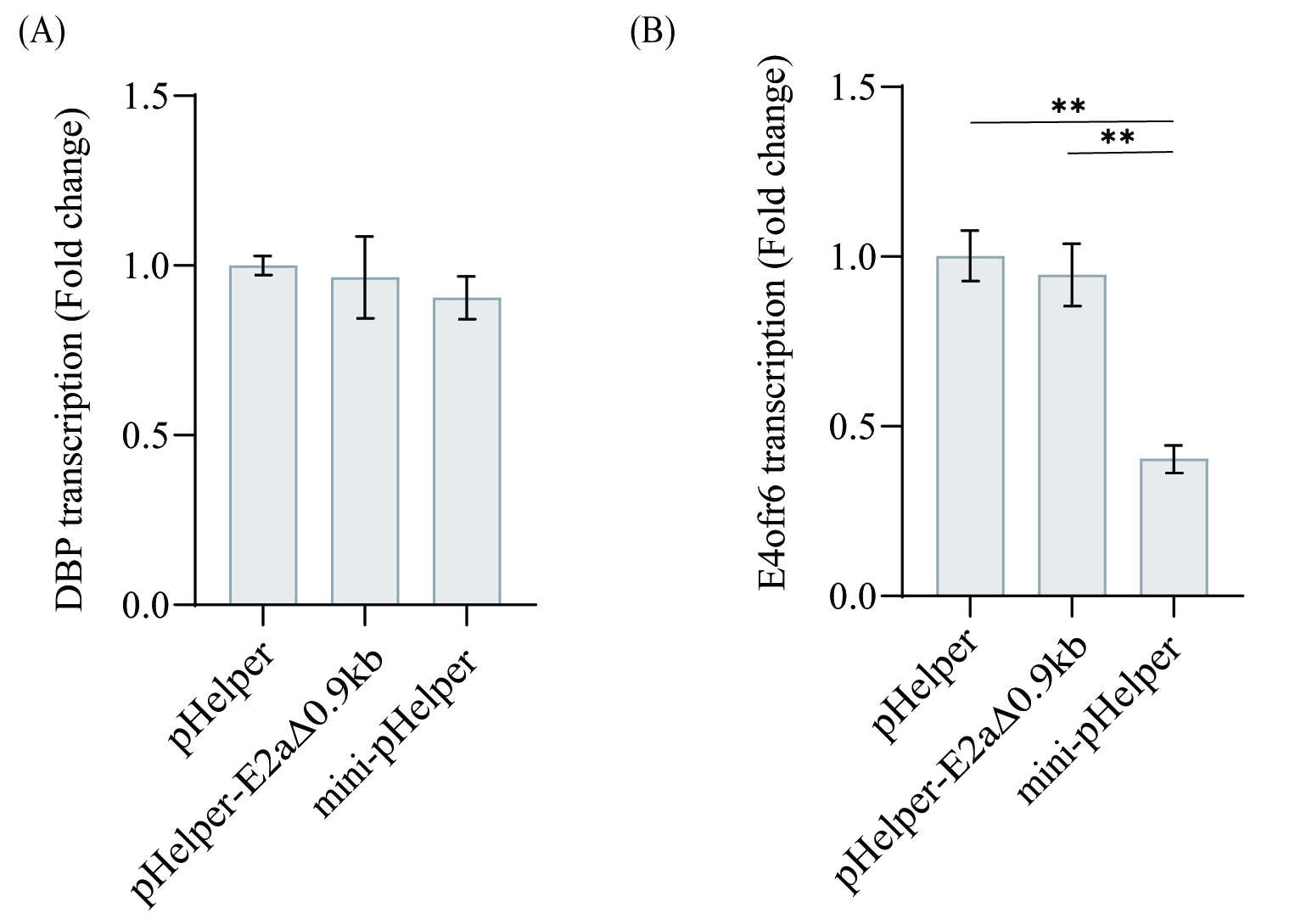


**Fig S2. Relative transcriptional activity of the mini-pHelper containing deletions within the E4 and E2a regions.**Quantitative analysis of mRNA expression levels for DBP (A) and E4orf6 (B) in HEK293 cells transfected with mini-pHelper, using GAPDH as the internal reference. Transcript levels are shown as fold change relative to the pHelper group. Data were calculated from three biological replicates. Statistical was analyzed by one-way ANOVA, * *p*< 0.05, ** *p*< 0.01.


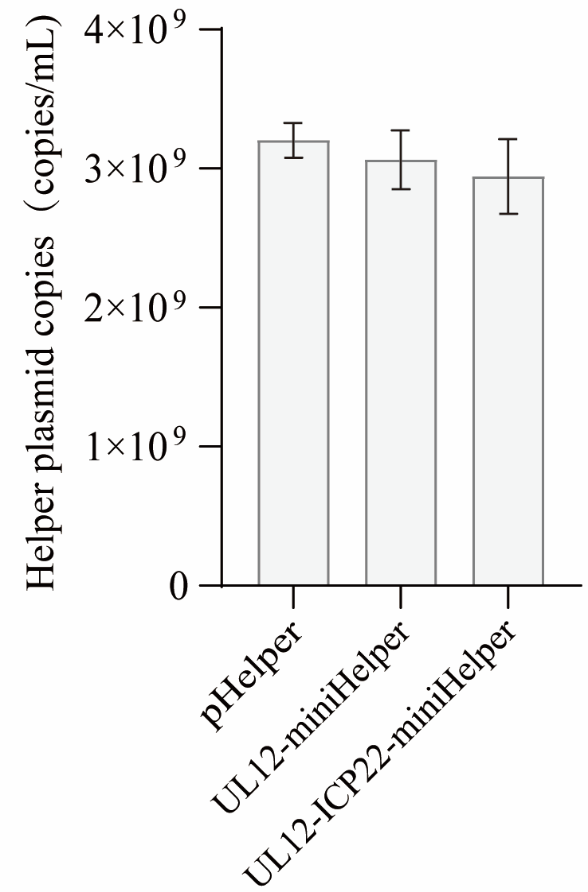


**Fig S3. Assessment of cellular uptake of constructed helper plasmid**

Assessment of helper plasmid uptake per mL in HEK293 co-transfected with pAAV-EGFP, pAAV-RC5, and pHelper, UL12-miniHelper or UL12-icp22-miniHelper.


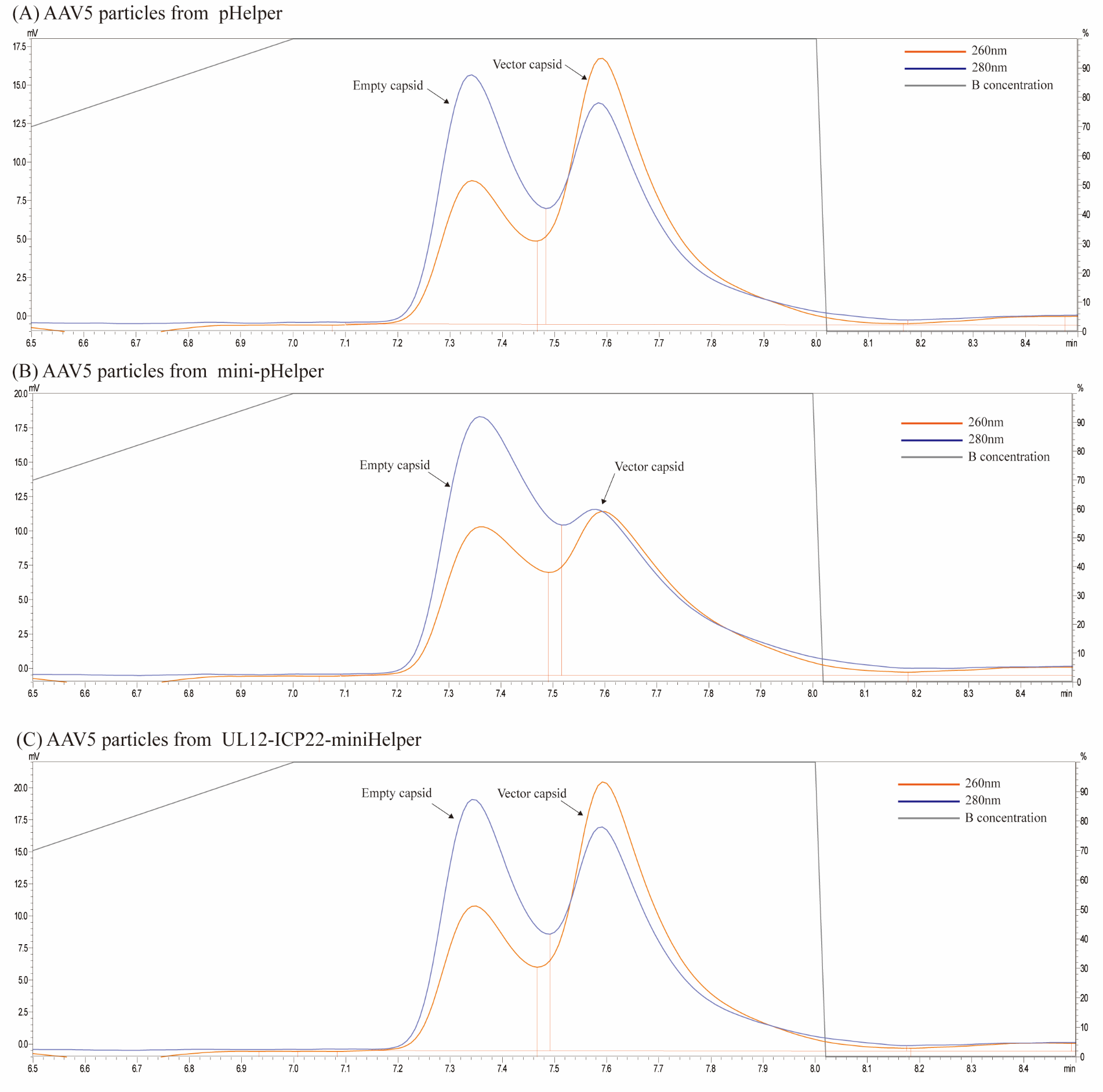


**Fig S4. Full/empty particle ratio analysis of AAV5 using afﬁnity chromatography**

Elution profiles of AAV5 particles produced using (A) pHelper, (B) mini-pHelper, or (C) UL12-ICP22-miniHelper. Particles puriﬁed by anion-exchange chromatography were applied to a CIMac Q 0.1 mL column and eluted using a linear gradient of NaCl from 0 to 500 mM.
